# Social Media Used and Teaching Methods Preferred by Generation Z Students in the Nursing Clinical Learning Environment: A Cross-Sectional Research Study

**DOI:** 10.3390/ijerph17218267

**Published:** 2020-11-09

**Authors:** M. Flores Vizcaya-Moreno, Rosa M. Pérez-Cañaveras

**Affiliations:** Department of Nursing, Faculty of Health Sciences, University of Alicante, 03690 San Vicente del Raspeig, Spain; rm.perez@ua.es

**Keywords:** clinical learning environment, nursing education, social media, teaching methods, Generation Z

## Abstract

Generation Z nursing students have a distinctive combination of attitudes, beliefs, social norms, and behaviors that will modify education and the nursing profession. This cross-sectional research study aimed to explore the social media use and characteristics of Generation Z in nursing students and to identify what were the most useful and preferred teaching methods during clinical training. Participants were Generation Z nursing degree students from a Spanish Higher Education Institution. A 41-item survey was developed and validated by an expert panel. The consecutive sample consisted of 120 students. Participants used social media for an average of 1.37 h (SD = 1.15) for clinical learning. They preferred, as teaching methods, linking mentorship learning to clinical experiences ($\bar{x}$ = 3.51, SD = 0.88), online tutorials or videos ($\bar{x}$ = 3.22, SD = 0.78), interactive gaming ($\bar{x}$ = 3.09, SD = 1.14), and virtual learning environments ($\bar{x}$ = 3, SD = 1.05). Regarding generational characteristics, the majority either strongly agreed or agreed with being high consumers of technology and cravers of the digital world (90.1%, *n* = 108 and 80%, *n* = 96). The authors consider it essential to expand our knowledge about the usefulness or possible use of teaching methods during clinical learning, which is essential at this moment because of the rapidly changing situation due to the Covid-19 pandemic.

## 1. Introduction

The majority of Millennials have moved to workplaces, and Generation Z has just arrived at Higher Education Institutions (HEI) around the world. Authors have referred to Generation Z as Gen Z, Gen-Xer, iGen, digital natives, net Generation, Zers, @generation, pluralist generation, Post-Millennials, Tweens, or eBay babies [1,2,3,4,5,6,7,8]. They are generally defined as the population born after 1995, despite some authors disagreeing on the exact cut-off date of this generation. Besides being authentic digital natives, they have a unique combination of attitudes, beliefs, social norms, and behaviors that will modify education and the nursing profession over the next few years [1,4,5,6,7,8,9,10].

Generation Z has been described as open-minded and more ethnically diverse than previous generations [2,3,4,5,6,7,8]. To establish relationships, they prefer social media to face-to-face communication, so it has been suggested that they could have poor social skills [4,5]. Previous studies notice that the excessive use of technology and little personal contact could affect social relations capacity and lead to isolation and an increase in mental illness in this population [1,5]. On the other hand, Gen Z is vigilant and concerned with physical, emotional, and financial stability [4,8]. They have a robust work ethic and believe that having superior education leads them to extensive work opportunities [6,10]. Gen Z is concerned about future employment, and they take fewer risks than previous generations. They prefer to feel satisfied in their job rather than gaining a high amount of money, so this generation would still choose a nursing degree [4,5,9,10].

Worldwide healthcare students use social media platforms for personal and educational purposes, mainly through mobile technology [11,12,13,14,15], and they expect their faculty to do the same [9]. HEI, healthcare centers, nursing faculty, and mentors should be aware of the increased use of social media by the new generations in order to update teaching methods to Millennials’ and Generation Z students’ needs and preferences [2,3,4,5,8,16].

In European Union countries, nursing education programs should include interesting updated information and incorporate mobile smart devices and social networks as part of teaching methods. These changes will help to capture nursing students’ attention in academic and practice environments [4,5,8,11,13,15]. A vast number of examples have been described in previous studies, such as video tutorials, YouTube^®^, Facebook^®^, WhatsApp^®^, Twitter^®^, concept mapping or infographics, hybrid or flipped classroom, storytelling, virtual learning environments, gaming, clicker technology or quizzes (Kahoot!^®^, Socrative^®^, Jeopardy^®^), blogs, simulations, role-playing, case study, jigsaw classroom, smartphone applications, and QR codes [1,5,6,8,11,16,17,18,19,20,21,22].

Previous studies describe some of the main variables that explain how Generation Z students learn. For example, they are electronic multitaskers, have an attention span of 8 s (12 s for Millennials) [6,8,9], and prefer active learning such as observation and experiential practice compared to traditional teaching methods [8,9]. They expect technology, not books [9], and select storytelling despite reading a book [8]. They may not pay much attention to detail and consider as valid the first information heard. Hampton and others [1] highlight a lack of understanding of the differences between the truth and opinions—that is, insufficient critical thinking capacity. They expect prompt feedback, prefer to communicate in short pieces of information (WhatsApp^®^, Twitter^®^, or Snapchat^®^) instead of using an email, or use images in their messages [6,7,8,9]. They may become frustrated if answers are not received instantly [8].

Nevertheless, there are still insufficient studies with a focus on the characteristics, social media use, and learning styles of Gen Z nursing students, and very few of them analyze the clinical education of this population [4,5,6,8,9]. Nowadays, the clinical training of nursing students supposes half of the minimum duration of the total nursing program [23]. European Union nursing students have to complete a total of 2300 clinical placement hours and must to acquire future professional nurses’ competences in complex clinical learning environments (e.g., hospitals, healthcare centers, or public health centers). Based on the model of mentoring nursing, the mentor is the person who mainly guides students’ learning process in this context and should update the traditional teaching methods and use social media platforms. Thus, knowing the learning preferences and characteristics of Generation Z students can help to nurse faculty and mentors to link better with them and increase learning motivation in the academical and clinical learning environments. Identifying how social media are used and which strategies and tools are most attractive for the clinical learning of Generation Z students, probably will allow us to involve and guide them successfully [4,6,9].

The purpose of this study was to explore the social media use and characteristics of Generation Z in nursing students and identify what are the most useful and preferred teaching methods and tools during clinical training from their point of view. Additionally, we wondered if the academic year of the students could be related to the type of social media used, teaching methods preferences, or their perception of Generation Z characteristics.

## 2. Materials and Methods

### 2.1. Design, Setting, and Participants

This descriptive cross-sectional survey study was carried on in a higher education institution in Spain. The target population were nursing degree students with previous experience in clinical practices and being an age less than or equal to 25 years as inclusion criteria.

### 2.2. Instrument and Procedure

A 41-item survey was developed based on the review of the scientific evidence on the subject published between 2000 and 2019 in the CINAHL (EBSCO), MEDLINE (Ovid) and ERIC databases. The questionnaire has 15 items about social media use [12], 14 items related to teaching methods during clinical practice [4,5] and 9 items about the grade of agreement with the characteristics attributed to the Generation Z [4,5]. Participants evaluated social media and teaching methods items through a frequency Likert scale (0 = never, 1 = rarely, 2 = sometimes, 3 = very often, 4 = always). The grade of agreement items was evaluated through a 5-point agreement Likert scale (strongly agree to strongly disagree). Demographic questions were also included (age in years, gender, and year on the nursing program). Before questionnaire distribution, a 10-member expert panel (seven clinical teachers, two university lecturers, and one master’s student) implemented the validity of the content of the instrument.

The target population were emailed a link to the survey developed using the Google Forms tool, with two reminders for completion with one-week apart. We have used email and several teaching rounds for the distribution of the access link to the questionnaire among the population under study. Data were collected between February and March 2020.

### 2.3. Data Analysis

Statistical analyses of the variables were conducted using the SPSS version 25 software (IBM Corporation, New York, NY, USA, 2017). We calculated frequencies, means, and standard deviations for descriptive analysis. Non-parametric correlation analysis was used to assess the correlation between demographic and the other variables studied. *p*-values < 0.05 were considered to be statistically significant.

### 2.4. Ethical Considerations

This study has adhered to the ethical requirements governing researcher and teacher responsibilities [24] (pp. 36–37) and was approved by the Ethics Committee of the University of Alicante (Expediente UA-2019-12-12). Participants received a letter by email providing information about the study with the invitation to participate and the access link to it. Their voluntary participation was interpreted as informed consent to participate.

### 2.5. Quality Appraisal

The authors followed the STROBE (STrengthening the Reporting of OBservational studies in Epidemiology) checklist for cross-sectional studies of EQUATOR (Enhancing the QUAlity and Transparency of Health Research) to provide quality to this study.

## 3. Results

### 3.1. Sample

A total of 586 nursing students in the undergraduate program were invited to participate in the study. The final consecutive sample consisted of 120 students that, according to the inclusion criterion were age less than or equal to 25 years. The response rate was 20.5%. The participants were 38 second-year (31.7%), 46 third-year (38.3%) and 36 fourth-year (30%) students, of whom 107 (89.2%) were female and 13 (10.8%) were male. They were aged between 19 and 25 years old ($\bar{x}$ = 20.77, SD = 1.57).

### 3.2. Social Media Use

Nursing students’ responses about the number of hours per day using social media for personal purpose or their clinical learning is shown in Table 1. Participants used social media an average of 2.56 h (SD = 1.74) for personal issues and 1.37 h (SD = 1.15) for clinical learning. Ten hours (*n* = 1; 0.8%) was the maximum of time per day for personal purposes, and 7 h (*n* = 1; 0.8%) for clinical learning.

Table 2 shows the students’ answers about the type of social media that they use for personal purposes or their clinical learning. The vast majority of participants affirmed that they always use WhatsApp (85.8%, *n* = 103) and always or very often (71.8%, *n* = 86) use Instagram for personal purposes. However, they said they never use or rarely use Facebook (69.2%, *n* = 83) for personal purposes. Generation Z students only highlighted (always or very often) the use of Google+/Currents (60%, *n* = 72) for their clinical learning.

The academic year of students was not significantly associated with the type of social media used for personal use. Only a weak correlation was found between the academic year of the student and the use of Twitter for clinical learning (Spearman’s rho = 0.21, *p* < 0.05).

### 3.3. The Extent of Use for Teaching Methods in Clinical Learning

Table 3 presents the students’ opinion about the extent of the usefulness or possible use of some teaching methods during clinical learning. The ones that were considered the best were linking mentorship learning to clinical experiences ($\bar{x}$ = 3.51, SD = 0.88), online tutorials or videos ($\bar{x}$ = 3.22, SD = 0.78), interactive gaming ($\bar{x}$ = 3.09, SD = 1.14), and virtual learning environments ($\bar{x}$ = 3, SD = 1.05). Using Facebook ($\bar{x}$ = 1.11, SD = 1.3) or Twitter ($\bar{x}$ = 1.3, SD = 1.4) were the least considered by participants.

A weak correlation was found between the academic year of the student and the use of Facebook for clinical practice knowledge update (Spearman’s rho = 0.33, *p* < 0.01), as well as with the use of interactive gaming (Spearman’s rho = 0.29, *p* < 0.01), Twitter (Spearman’s rho = 0.26, *p* < 0.01), and flipped classroom (Spearman’s rho = 0.23, *p* < 0.01). 

### 3.4. Degree of Agreement with the Generation Z Characteristics

Participants’ responses around the grade of agreement with the characteristics attributed to Generation Z [5] are shown in Table 4. The majority of students either strongly agreed or agreed with being high consumers of technology and cravers of the digital world (90.1%, *n* = 108); open-minded, diverse, and comfortable with diversity (80%, *n* = 96); and increased risk of isolation, anxiety, insecurity, and depression (62.5%, *n* = 75). About half of them disagreed or strongly disagreed with having underdeveloped social and relationship skills (50%, *n* = 60) or conducting sedentary activism (45%, *n* = 54). The academic year of students was not significantly associated with the participant’s perception of Generation Z characteristics.

## 4. Discussion

In this study, the majority of participants affirmed the use of social media around the double-time for personal purposes than for clinical education. The majority of them pointed to WhatsApp and Instagram for personal purposes and Google Currents (Google+) for clinical learning. These Gen Z students preferred the following clinical learning methods: linking mentorship learning to clinical experiences, use of online tutorials or videos, interactive gaming, and virtual learning environments. They also strongly agreed or agreed with being high consumers of technology and cravers of the digital world, open-minded, diverse, and comfortable with diversity, and with increased risk for isolation, anxiety, insecurity, and depression. However, they disagreed or strongly disagreed with having underdeveloped social and relationship skills or performing sedentary activism. We only found several weak correlations between the academic year of the students with the use of Twitter as social media and the preference of teaching methods as Facebook and Twitter for knowledge update, interactive gaming, and the flipped classroom.

Consistent with Duke and colleagues [12], our study found than half of Generation Z students spent 1–3 h per day on social media for personal use. Nevertheless, Shato and Erwin [7,8] found spending around 9 h per day using smartphones for digital communication. During clinical practice, smartphones are the most popular device use for social media consumption [11,14,15]. Cho and Lee [14] warned of the risk of its use as facilitate students and healthcare professional distraction and could affect patient safety. 

Regarding the type of social media use, previous studies identified as the most popular Facebook, Twitter, and YouTube [3,12,15,18,19,21]. Oducado [18] studied the educational use of Facebook by Gen Z nursing students analyzing their perception and satisfaction, finding that the majority of them use it to communicate (91.15%), collaborate (87.61%), and share academic sources or materials (85.84%), and they generally were highly satisfied (77%). Nevertheless, easy distraction with other content was identified as the central trouble (81.42%) in Facebook’s use for educative purposes. However, the majority of our students said they never used or rarely used Facebook. A probable explanation of this is that previous studies explored the intensity and typology of social media interaction but disregarded students’ generational characteristics, mixing Millennials and Gen Z students.

Pimmer [19] found that WhatsApp was of high use and perceived as useful by nursing students during clinical placement. They explained that connecting nursing students with other significant people in the clinical context as peers, mentors, lectures, or other students is WhatsApp’s success key. In our study, the majority of Generation Z students highlighted the use of WhatsApp and Instagram.

Using social media in clinical training can provide nursing faculty and mentors the context for discussing with the students and colleagues about patient confidentiality and privacy, nursing ethics, public image and privacy, data protection, and also students can increase awareness about their profession (eProfessionalism) [6,8,12,21]. Moreover, during clinical nursing training social media can be an aid to build and support social and educative relationships between students and lecturers, letting the feedback and emotional support that nursing students need [21,25]. Previous evidence suggests that motivational level of students seems to influence students’ participation in social media for educative purpose, mainly when evaluation time is coming [21]. Besides, some of the nursing students feel confident in using social networks for deepening their understanding of a subject and checking if they have made any progress. However, have been identified some negative factors that are interfering with clinical learning as the operative problems of social media platforms and the nursing students’ distractions while being online [21].

The generational-diverse workforce in healthcare settings, called Baby Boomers, Generation X, Generation Y, or Millennials [26] and Generation Z students [4] have to avoid conflict and create links between generations to develop dynamic learning environments [7,27]. Regarding with Generation Z’s learning characteristics, they preferred practical and relevant information and learning what is visually based, exciting, immediate, engaging, individuals based or technologically advanced, in faculty or clinical learning environments [5]. Hampton and colleagues [1] studied the learning preferences and engagement level in a sample of Gen Z nursing students, finding that a lecture with audience response (clickers) was the most preferred and engaging methods, and assigned reading was the least one preferred. For that reason, authors suggest combining innovative lectures (e.g., use of visual images or videos) with the use of simulation and case studies to stimulate engagement and learning levels.

We cannot explain these generational characteristics focusing only on social and cultural values or beliefs. Zarra [28] describes how these students’ brains become wired to understand complex visual images, making optical approaches to teaching more effective than others. For example, Generation Z students do not learn by reading and listening to a PowerPoint^®^ presentation. They learn by observation and experiential practice [7,8], so that YouTube videos are very popular and valuable in teaching concepts to Gen Z students [8].

Regarding these clinical teaching methods, Attenborough and Abbott [17] reported that faculty perceived storytelling as valid for linking theory and practice, and rise empathy, understanding, and nursing identity in nursing students. Notwithstanding, our Gen Z students preferred linking mentorship learning to clinical experiences, online tutorials/videos, interactive gaming, or virtual learning environments to storytelling.

Consistent with our findings, previous studies found that interactive gaming enhances experiential learning, encourage critical thinking, making the learning fun and increasing students’ involvement and motivation [8,16]. McEnroe-Pettiete and Farris [16] suggested developing interactive clinical stations where students capture with their phones correct nursing techniques and methods, providing them with video evidence. Nursing students should get points of the assigned activity. We should keep in mind that disruptive, stress, or embarrassment feelings in students could arise [16].

The generational characteristics used in the present study are attributes identified in previous literature, and not all Generation Z has to be aligned with these social stereotypes [4]. Our nursing students strongly agreed or agreed with some of Generation Z’s characteristics and disagreed or strongly disagreed with others. We have not found similar studies to make a comparison regarding the degree of identification of students with the nine attributes of Generation Z defined by Chicca and Shellenbarger [4].

The main strength of this study is that it shows a picture of the prevalence of the outcomes of interest—social media use, preferred teaching methods for clinical learning, and level of identification with Generation Z attributes—in a sample of nursing students. This study limitations include convenience sampling and restriction to one HEI, which could affect its generalizability. Data were collected previous to the Covid-19 pandemic, and probably we could obtain differing results if another timeframe had been chosen.

## 5. Conclusions

The authors consider it essential to expand our knowledge about the usefulness or possible use of teaching methods during clinical learning, which can be essential at this moment because of the rapidly changing situation due to the Covid-19 pandemic.

In the current study, the participants used social media an average of 1.37 h for clinical learning and around double that time for personal purposes. For the clinical learning environment, they preferred teaching methods such as linking mentorship learning to clinical practice, online tutorials or videos, interactive gaming, and virtual learning environments. Regarding generational characteristics, the majority either strongly agreed or agreed with being high consumers of technology and cravers of the digital world, and with being open-minded, diverse, and comfortable with diversity. We would like to highlight as a negative issue that their level of agreement with the characteristic of having increased risk of isolation, anxiety, insecurity, and depression is unfortunately high.

The Generation Z nursing students’ characteristics and their preferences regarding educational tools and strategies should be taken into account in designing clinical learning environments to enhance their quality and the satisfaction between students, nursing faculty, and mentors. Future studies should investigate the possible efficacy of these new teaching methods from a reflective perspective and include consequences such as data protection or patient safety.

## Figures and Tables

**Table 1:** Frequencies of participant responses about the number of hours per day using social media for personal purposes and clinical learning (*n* = 120).

Time	Personal		Clinical Learning	
Hours	*n*	%	*n*	%
<0.5	6	5	26	21.5
0.5–1	26	21.7	59	49.2 *
1–2	34	28.3 *	17	14.2
2–3	29	24.2	12	10
3–4	10	8.3	3	2.5
4–5	7	5.8	2	1.7
>5	8	6.6	1	0.8

* Higher scores frequencies of participant response.

**Table 2:** Frequencies of participant responses about the type of social media used for personal purposes or for their clinical learning.

Type of Social Media	Personal Use ^1^							Clinical Learning ^1^						
	Mean (SD)	Never	Rarely	S.T.	Very Often	Always	N/A	Mean (SD)	Never	Rarely	S.T.	Very Often	Always	N/A
YouTube	1.96 (1.33)	24 (20)	15 (12.5)	29 (24.2)	31 (25.8)	11 (11.7)	7 (5.8)	1.91 (1.22)	24 (20)	9 (7.5)	41 (34.2)	31 (25.8)	8 (6.7)	7 (5.8)
Google Currents/Google+	1.57 (1.54)	42 (35)	16 (13.3)	14 (11.7)	18 (15)	18 (15)	12 (10)	2.55 * (1.59)	26 (21.7)	4 (3.3)	10 (8.3)	26 (21.7)	46 (38.3)	8 (6.7)
WhatsApp	3.78 * (0.65)	2 (1.7)	-	3 (2.5)	12 (10)	103 (85.8)	-	1.92 (1.41)	28 (23.3)	14 (11.7)	23 (19.2)	29 (24.2)	16 (13.3)	10 (8.3)
Facebook	0.85 (1.22)	65 (54.2)	18 (15)	15 (12.5)	6 (5)	7 (5.8)	9 (7.5)	0.22 (0.66)	91 (75.8)	9 (7.5)	2 (1.7)	2 (1.7)	1 (0.8)	15 (12.5)
Twitter	1.49 (1.66)	54 (45)	9 (7.5)	12 (10)	12 (10)	24 (20)	9 (7.5)	0.41 (0.89)	83 (69.2)	12 (10)	5 (4.2)	6 (5)	1 (0.8)	13 (10.8)
Instagram	2.98 * (1.16)	7 (5.8)	7 (5.8)	16 (13.3)	37 (30.9)	49 (40.9)	4 (3.3)	1.05 (1.14)	48 (40)	14 (11.7)	23 (19.2)	15 (12.5)	8 (6.7)	12 (10)

^1^ Never = 0; Always = 4. S.T. = sometimes. N/A = no answer. *n* (%). * Higher scores of social media used for personal or clinical learning purposes.

**Table 3:** Students’ opinion about the usefulness or possible usefulness of teaching methods in clinical learning.

Teaching Methods	Frequency of Possible Use ^1^						
	Mean (SD)	Never	Rarely	Sometimes	Very Often	Always	N/A
Linking mentorship learning (mentor/nurse-tutor) to clinical experiences	3.51 (0.88)	2 (1.7)	5 (4.2)	10 (8.3)	28 (23.3)	69 (57.5)	6 (5)
Online tutorials or videos for use in clinical skills training	3.22 (0.78)	-	3 (2.4)	17 (14.2)	50 (41.7)	50 (41.7)	-
Interactive gaming, such as Jeopardy, Kahoot!, and Socrative	3.09 (1.14)	7 (5.8)	4 (3.4)	18 (15)	31 (25.8)	58 (48.3)	2 (1.7)
Virtual learning environments	3 (1.05)	3 (2.5)	10 (8.3)	17 (14.2)	43 (35.8)	46 (38.4)	1 (0.8)
Readings that can be completed on tablets and/or smartphones	2.99 (1.14)	6 (5)	9 (7.5)	15 (12.5)	39 (32.5)	50 (41.7)	1 (0.8)
Tell a clinical story	2.85 (1.04)	4 (3.4)	6 (5)	32 (26.7)	37 (30.8)	38 (31.7)	3 (2.4)
Use problem-based learning	2.85 (1.02)	3 (2.4)	9 (7.5)	24 (20)	43 (35.8)	34 (28.3)	7 (5.8)
Use case studies	2.75 (1.03)	3 (2.4)	10 (8.3)	32 (26.7)	40 (33.3)	32 (26.7)	3 (2.4)
Students videotape themselves giving and taking nursing reports	2.59 (1.35)	13 (10.8)	13 (10.8)	23 (19.2)	27 (22.5)	40 (33.3)	4 (3.4)
Use flipped classroom applied to clinical learning	2.53 (1.35)	9 (7.5)	14 (11.7)	19 (15.8)	25 (20.9)	29 (24.1)	24 (20)
Use concept mapping applied to clinical learning	2.49 (1.21)	9 (7.5)	13 (10.8)	27 (22.5)	34 (28.3)	25 (20.9)	12 (10)
Virtual group work	2.28 (1.23)	11 (9.1)	21 (17.5)	31 (25.8)	31 (25.8)	22 (18.3)	4 (3.4)
Twitter for clinical practice knowledge update	1.3 (1.4)	54 (45)	14 (11.7)	17 (14.2)	19 (15.8)	11 (9.1)	5 (4.2)
Facebook for clinical practice knowledge update	1.11 (1.3)	57 (47.4)	17 (14.2)	17 (14.2)	19 (15.8)	5 (4.2)	5 (4.2)

^1^ Never = 0; Always = 4. N/A: no answer. n (%).

**Table 4:** Students’ degree of agreement with the characteristics attributed to Generation Z.

Characteristics	Degree of Agreement ^1^					
	Mean (SD)	Strongly Agree	Agree	Undecided	Disagree	Strongly Disagree
High consumers of technology and cravers of the digital world	4.3 (0.69)	49 (40.9)	59 (49.2)	11 (9.1)	-	1 (0.8)
Open-minded, diverse, and comfortable with diversity	4.12 (0.94)	47 (39.2)	49 (40.8)	17 (14.1)	4 (3.4)	3 (2.5)
Increased risk for isolation, anxiety, insecurity, and depression	3.65 (1.03)	23 (19.2)	52 (43.3)	29 (24.1)	11 (9.2)	5 (4.2)
Cautious and concerned with emotional, physical, and financial safety	3.61 (1.08)	26 (21.7)	44 (36.7)	31 (25.8)	14 (11.7)	5 (4.1)
Pragmatic	3.58 (0.82)	13 (10.8)	53 (44.2)	46 (38.3)	6 (5)	2 (1.7)
Lack of attention span, desiring convenience and immediacy	3.26 (1.1)	16 (13.3)	37 (30.8)	36 (30)	24 (20.1)	7 (5.8)
Individualistic	3.2 (1.09)	16 (13.3)	31 (25.8)	40 (33.3)	27 (22.6)	6 (5)
Sedentary activism	2.81 (1.14)	8 (6.6)	29 (24.2)	29 (24.2)	40 (33.3)	14 (11.7)
Underdeveloped social and relationship skills	2.58 (1.0)	5 (4.2)	15 (12.5)	40 (33.3)	45 (37.5)	15 (12.5)

^1^ 5-point Likert scale (5 = strongly agree; 1 = strongly disagree). *n* (%).

## References

[1] Hampton D., Welsh D., Wiggins A.T. (2020). Learning Preferences and Engagement Level of Generation Z Nursing Students. Nurse Educ..

[2] Mohr E.S., Mohr K.A. (2018). The ABCs of the XYZs: Adding a Critical Dimension to Contemporary Teacher Education. J. Educ. Train. Stud..

[3] Mohr K.A.J., Mohr E.S. (2017). Understanding Generation Z Students to Promote a Contemporary Learning Environment. J. Empower. Teach. Excell..

[4] Chicca J., Shellenbarger T. (2018). Generation Z: Approaches and Teaching-Learning Practices for Nursing Professional Development Practitioners. J. Nurses Prof. Dev..

[5] Chicca J., Shellenbarger T. (2018). Connecting with Generation Z: Approaches in Nursing Education. Teach. Learn. Nurs..

[6] Hampton D.C., Keys Y. (2016). Generation Z students: Will they change our nursing classrooms?. J. Nurs. Educ. Pract..

[7] Shatto B., Erwin K. (2017). Teaching Millennials and Generation Z: Bridging the generational divide. Creat. Nurs..

[8] Shatto B., Erwin K. (2016). Moving on from Millennials: Preparing for Generation Z. J. Contin. Educ. Nurs..

[9] Williams C.A. (2019). Nurse Educators Meet Your New Students: Generation Z. Nurse Educ..

[10] Hampton D., Welsh D. (2019). Work Values of Generation Z Nurses. J. Nurs. Adm..

[11] Lee H., Min H., Oh S.M., Shim K. (2018). Mobile technology in undergraduate nursing education: A systematic review. Healthc. Inform. Res..

[12] Duke V.J.A., Anstey A., Carter S., Gosse N., Hutchens K.M., Marsh J.A. (2017). Social media in nurse education: Utilization and E-professionalism. Nurse Educ. Today.

[13] Mackay B.J., Anderson J., Harding T. (2017). Mobile technology in clinical teaching. Nurse Educ. Pract..

[14] Cho S., Lee E. (2016). Distraction by smartphone use during clinical practice and opinions about smartphone restriction policies: A cross-sectional descriptive study of nursing students. Nurse Educ. Today.

[15] Strandell-Laine C., Stolt M., Leino-Kilpi H., Saarikoski M. (2015). Use of mobile devices in nursing student-nurse teacher cooperation during the clinical practicum: An integrative review. Nurse Educ. Today.

[16] McEnroe-Petitte D., Farris C. (2020). Using Gaming as an Active Teaching Strategy in Nursing Education. Teach. Learn. Nurs..

[17] Attenborough J., Abbott S. (2020). Using storytelling in nurse education: The experiences and views of lecturers in a higher education institution in the United Kingdom. Nurse Educ. Pract..

[18] Oducado R.M.F. (2019). Gen Z Nursing Students’ Usage, Perception and Satisfaction with Facebook for Educational Purposes: Tool for Learning or Distraction. Indones. Nurs. J. Educ. Clin..

[19] Pimmer C., Brühlmann F., Odetola T.D., Dipeolu O., Gröhbiel U., Ajuwon A.J. (2018). Instant messaging and nursing students’ clinical learning experience. Nurse Educ. Today.

[20] Toothaker R. (2018). Millennial’s perspective of clicker technology in a nursing classroom: A Mixed methods research study. Nurse Educ. Today.

[21] O’Connor S., Jolliffe S., Stanmore E., Renwick L., Booth R. (2018). Social media in nursing and midwifery education: A mixed study systematic review. J. Adv. Nurs..

[22] Xu J. (2016). Toolbox of teaching strategies in nurse education. Chin. Nurs. Res..

[23] Directive 2013/55/EU of the European Parliament and of the Council Office Journal of the European Union no l 354/132. https://eur-lex.europa.eu/eli/dir/2013/55/oj.

[24] Cormark D.F.S. (1996). The Research Process: In Nursing.

[25] Sigalit W., Sivia B., Michal I. (2017). Factors Associated with Nursing Students’ Resilience: Communication Skills Course, Use of Social Media and Satisfaction with Clinical Placement. J. Prof. Nurs..

[26] Bishop P., Wackler T. (2017). Education strategies for generation Y. J. Contin. Educ. Nurs..

[27] Hart S. (2017). Today’s Learners and Educators: Bridging the Generational Gaps. Teach. Learn. Nurs..

[28] Zarra E.J. (2017). The Entitled Generation: Helping Teachers Teach and Reach the Minds and Hearts of Generation Z.

